# Dog Stick Chewing: An Overlooked Instance of Tool Use?

**DOI:** 10.3389/fpsyg.2020.577100

**Published:** 2021-01-13

**Authors:** James Brooks, Shinya Yamamoto

**Affiliations:** ^1^Wildlife Research Center, Kyoto University, Kyoto, Japan; ^2^Institute for Advanced Study, Kyoto University, Kyoto, Japan

**Keywords:** *Canis lupus familiaris*, dog cognition, stick chewing, teething, object manipulation, animal tool use

## Abstract

Tool use is a central topic in research on cognitive evolution and behavioral ecology in non-human animals. Originally thought to be a uniquely human phenomenon, many other species have been observed making and using tools for a variety of purposes, starting with Goodall’s (1964) groundbreaking work with chimpanzees in Gombe. Despite the frequent attention and great research interest in animal tool use, and ubiquity of the behavior, we argue here that chewing sticks by dogs (and other animals) should be included as a case of tool use. We discuss alternate possible explanations and then propose several testable predictions regarding this hypothesis. We suggest that tool use may be more common than is often assumed and that many cases of animal tool use may be overlooked.

## Introduction

Although it was once thought to be unique to humans, a growing number of species have now been observed using tools (Shumaker et al., 2011). Cognitive scientists and behavioral ecologists have devoted great research attention to tool use behavior in order to shed light on the evolution of physical reasoning skills and in some cases complex foraging strategies. Tool use is often defined as

“The external employment of an unattached or manipulable attached environmental object to alter more efficiently the form, position, or condition of another object, another organism, or the user itself, when the user holds and directly manipulates the tool during or prior to use and is responsible for the proper and effective orientation of the tool” (Shumaker et al., 2011, p. 5).

This definition came as a review and update to Beck’s (1980) definition, which has been called “the current standard” (Amant and Horton, 2008, p. 1199) until the revision, which likewise has been called “the closest thing to a standard definition” (McGrew, 2013, p. 2). Other academics have proposed modifications, simplifications, or alternatives for how to identify tool use, but Shumaker et al.’s (2011) definition remains widely used. Alternate formulations include, for example, Van Lawick-Goodall’s (1970, p. 195) early, more abstract definition characterizing tool use as “the use of an external object as a functional extension of mouth or beak, hand or claw, in the attainment of an immediate goal,” Matsuzawa’s (2001. p. 6) more inclusive definition describing tool use as “a set of behaviors utilizing a detached object to obtain a goal that is adaptive in the biological sense,” or Amant and Horton’s (2008, p. 1203) more recent formulation breaking the concept into two kinds, either through “(1) altering the physical properties of another object, substance, surface or medium (the target, which may be the tool user or another organism) via a dynamic mechanical interaction, or (2) mediating the flow of information between the tool user and the environment or other organisms in the environment,” but today, Shumaker et al.’s (2011) definition remains the standard in the literature. Tool use is relatively rare but can be found across many orders, most commonly in Passeriformes and Primates, and most often in social, feeding, or self-maintenance contexts (Shumaker et al., 2011). Classic examples of tool use in non-human animals include the complex repertoire of rocks and sticks used by chimpanzees (*Pan troglodytes*) in extractive foraging, such as hammers to break nuts and twigs to “fish” for insects (e.g., Goodall, 1964; Boesch and Boesch, 1990; Inoue-Nakamura and Matsuzawa, 1997; Whiten et al., 1999), several self-maintenance behavior in elephants (*Loxodonta africana* and *Elephas maximus*), such as branches for fly swatting and small sticks for self-scratching (Douglas-Hamilton and Douglas-Hamilton, 1978; Chevalier-Skolnikoff and Liska, 1993; Hart and Hart, 1994), and ever-increasing examples of tool use in birds, including the insect fishing probes of New Caledonian crows (*Corvus moneduloides*) (Hunt, 1996; Hunt and Gray, 2004) and recently sticks for self-scratching in Atlantic puffins (*Fratercula arctica*) (Fayet et al., 2020). Shumaker et al. (2011) also identify several borderline cases such as the use of objects fixed in the environment and bird nest construction, where intuitions are split and definitions can have trouble confirming or denying their status as tool use. The authors, noting this, questioned the view that tool use is a simple binary of either present or absent, but provide their definition as a way to move forward and identify cases despite the lack of an absolute separation between tool use and non-tool use. In this paper, we argue that chewing sticks, a common but possibly underappreciated behavior in domestic dogs (*Canis lupus familiaris*) and possibly other species, qualifies as tool use under many current definitions, of which we will focus on Shumaker et al.’s (2011) definition. We further suggest that there may be many similar examples of tool use in contexts and species that have not yet been identified as such.

## Dogs Chewing Sticks: Tool Use?

**FIGURE 1:**
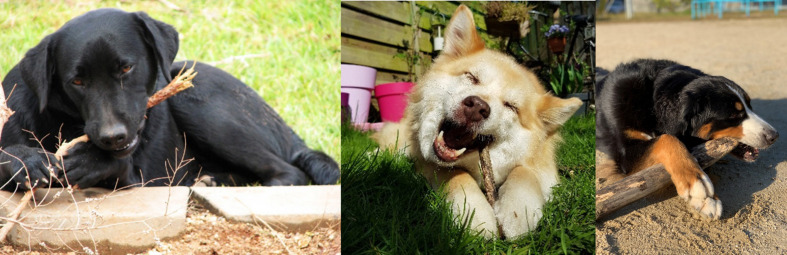
Examples of possible tool use cases, where the dog holds a detached environmental object possibly in order to reduce tooth pain or to clean teeth or gums. The first two images come from public databases and the third was taken by SY.

Although dogs chewing sticks is a behavior observed by almost all dog owners, no scientific papers have made the case that it may represent an instance of tool use. Most frequently, teething puppies (the stage of development where adult teeth erupt, involving irritation, and discomfort) will chew on a variety of objects, possibly to soothe their tooth pain. This is likely parallel to recommendations to provide hard objects for chewing to teething children in order to relieve soreness (e.g., Ashley, 2001). Many puppies will seek out sticks, and are often observed holding the stick between their front paws to stabilize it. Under Shumaker et al.’s (2011) definition, these dogs are using tools. They are using an object unattached to the environment (a stick), to modify a condition of the user itself (minimizing their own tooth pain or possibly cleaning teeth), and hold the tool during use, responsible for the proper and effective orientation of the tool (positioning and holding the stick between their front paws in order to reach specific teeth). Dogs often alternate between sides of their mouth, and re-center and stabilize the stick with their paws seemingly in order to more effectively target some teeth or apply pressure to specific areas of their mouth. Although detailed studies are needed, discussed in-depth below, in many cases, the dogs seem to be chewing in order to gain the desired sensation through the chewing, as opposed to attempting to break apart the stick most efficiently. The dogs will then spit out wood fragments that are removed and continue down the stick until little remains (see Supplementary Material for examples). Even in the case of adult dogs, where it is unlikely tooth pain explains all cases, there is no reason the sensation gained through chewing should not count as a condition they seek. It is also possible that chewing sticks yields benefits for tooth and gum health, which implies that using sticks in such a manner has a direct role on tooth-cleaning. The sensation of crunching a stick between their teeth can be analogized to the desire to scratch an itch, which is widely regarded as tool use in several species (see below). See Figure 1 for example cases and Supplementary Material for further examples and videos.

The lack of attention to dog stick chewing as tool use is intriguing, possibly owing to being seen as a familiar, unremarkable activity, or to dogs not traditionally being viewed as sufficiently cognitively complex, especially in physical reasoning abilities. The form of dog stick chewing may also be sufficiently different from “classic” tool use where tools are often grasped by forelimbs, are applied to the external environment or body surface, or are more intuitively used like an extended body part as in Van Lawick-Goodall’s (1970) definition. Adult human researchers additionally may recognize cases of tool use they can identify with, such as opening nuts, transferring food to their mouths, or scratching an itchy but hard to reach part of their body more readily than cases without such direct analogies to themselves. In any case, we argue here that despite the lack of research attention, dog stick chewing may represent an important and insightful case of tool use warranting future study.

## Possible Challenges

Although reducing tooth pain or cleaning teeth are the most obvious possibilities for the immediate function of the behavior, other possibilities must be examined closely. One alternate possibility is that chewing sticks is a behavioral substitution for bones that would be chewed by wild dogs and wolves. This explanation is possible but does not effectively rule out the behavior as tool use if indeed stick chewing behavior is elicited by and mitigates tooth pain or cleans teeth. Even in the case that the behavior is not observed when canids are given bones to chew, due to bones filling the same purpose in a feeding context, the use of external objects, manipulated properly, to cause a change in the condition in the user, qualifies the behavior as tool use regardless of whether it can be achieved through feeding in other contexts. An interesting question that arises from classifying dog stick chewing as tool use is how to classify cases where similar functional benefit and motor patterns are used but in addition nutrition is gained through eating parts of the possible tool, for example, wolf pups chewing on bones or dogs given (edible) rawhide chews designed for tooth and gum health. Dogs chewing without eating sticks cannot be readily excluded as a case of legitimate tool use. That said, the other cases mentioned, as well as other predators foraging on animal skin and bones (if they impact tooth and gum health), pose a challenge to straightforward classifications. Perhaps they can be grouped with other borderline cases such as the use of environmentally fixed objects in ways that otherwise would be considered tool use, or nest building behavior, which, as Shumaker et al. (2011) note, likely are not entirely different kinds of behavior but still have trouble fitting into standard definitions. A related alternate hypothesis is that dogs merely eat sticks and chew them in the process. This is usually not the case (see Supplementary Material for examples), but detailed observation should be conducted to fully eliminate this possibility.

Stick chewing may also be challenged as simply a play-related behavior in some cases, similar to object-oriented play in other species, which is not widely considered tool use in the absence of other obvious functional roles (Shumaker et al., 2011). The key difference here is the direct and immediate functional role of chewing sticks in dog tooth pain and potentially in tooth cleaning, which, if reliably demonstrated, would indicate the behavior is functional rather than “merely” playful. Shumaker et al. (2011) recognize some object-oriented play as tool use (highlighted in cases where objects are used to trigger or enhance social play^1^), and the interaction between play and tool use, especially the possible progression from object-oriented exploratory play toward functional tool use, has been highlighted by others (e.g., Cenni et al., 2020). Like tool use, play has long remained difficult to define precisely, though there are some common elements. Most notably, many definitions emphasize the lack of a functional role (e.g., Bekoff and Byers, 1981; Millar, 1981; Smith, 1982; Martin and Caro, 1985; Burghardt, 2005), which runs contrary to the characterization of dog stick chewing as play if the direct benefits to the animal are demonstrated conclusively. Some researchers have additionally employed structure–function interfaces (Pellis and Pellis, 1998) as a means of distinguishing play from other behaviors, including between object-oriented play and tool use (e.g., Pelletier et al., 2017; Cenni et al., 2020). These approaches may also be invoked in the study of stick chewing to identify functional from non-functional patterns and examine those that may be best identified as play rather than tool use. A direct and immediate benefit to the animal precludes characterizing the behavior as play by many definitions, though we fully recognize the challenges in defining play. We therefore do not claim that stick chewing can never be characterized as play, but instead emphasize that regardless of whether stick chewing can in some cases be classified play, the overt behavior fits most major definitions of tool use and thus can be considered as such. While we do not here propose a detailed substantive position on when play can and cannot include tool use, we emphasize that play and tool use need not be mutually exclusive. The same challenge can arise in other instances of animal tool use, where, despite a functional role, the behavior may be elicited directly through playful inclination. Further research, discussed in more detail below, should therefore be conducted on alternate and complementary explanations and characterizations of stick chewing in dogs. Still, the functional roles are clear reasons to consider the possibility that dog stick chewing in some contexts is a case of tool use as opposed in all cases being reducible to mere object-oriented play.

In a similar vein, stick chewing may alternatively represent a behavior more analogous to fidgeting by manipulating an object. We consider this hypothesis unlikely, as human research indicates fidgeting is indicative of activity overflow when physical movement is constrained by the focal task (Mehrabian and Friedman, 1986). Dogs are typically not constrained in such ways or engaged in other tasks during bouts of stick chewing and therefore likely are not exhibiting fidgeting behavior. In addition, fidgeting and tool use may not be mutually exclusive, for instance, some might consider fidget toys a kind of tool. Still, future research investigating whether dogs perform stick chewing more following focused activity, during periods of high stress, or in more closed environments would be interesting and useful for better understanding the specific elicitors of stick chewing in dogs.

Another important question to consider before accepting dog stick chewing as tool use is if in any cases of stick chewing, they have a goal in any sense. Many definitions of tool use, but notably not Shumaker et al.’s (2011), include the term “goal,” though this can be difficult or impossible to directly evaluate on the basis of external and measurable spontaneous behavior alone. If categorizing a behavior as tool use is based only on the observable properties of an object-oriented behavior, then a direct and immediate benefit to the animal that results from the use of a detached object, manipulated properly, is more valuable in identifying tool use than debates about whether the internal state of the animal represents a goal proper. The emphasis on a direct and immediate benefit, as opposed to an internal mental representation, may make classifications more straightforward by relying instead upon external, measurable behavior. Further, some tool use is thought to result from hardwired, inflexible behavioral specializations (Call, 2013), for which the term goal is even more difficult to interpret. If the definition is instead focused on a direct and immediate benefit to the animal, this view has no difficulty in accepting these inflexible and instinctive cases of tool uses. Replacing the term “goal” with a direct and immediate benefit to the animal in some definitions of tool use generates a more valuable and quantifiable definition by which to evaluate putative cases and more easily account for instinctive tool use and tool use involved in play.

**FIGURE 2:**
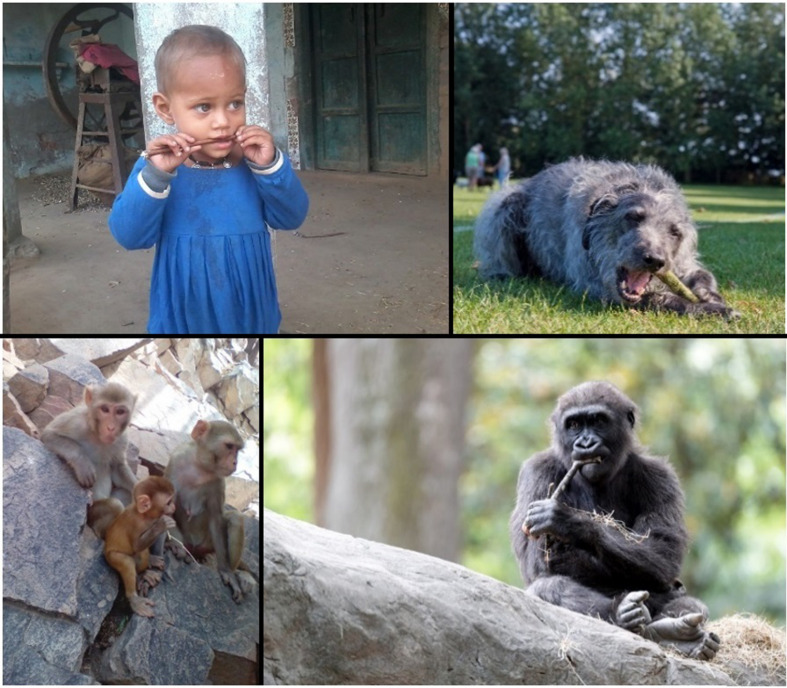
Images of stick chewing in several species. Only in humans is this considered tool use. All images come from public databases. Clockwise from top left: Human (*Homo sapiens*), Domestic dog (*Canis lupus familiaris*), Western lowland gorilla (*Gorilla gorilla*), and Rhesus macaque (*Macaca mulatta*).

If chewing is caused by, at least in some cases, a pain in the teeth and some inclination to chew sticks given that pain, or occurs after consuming food that is likely to get stuck in their teeth, this direct and immediate function of the behavior should qualify it as tool use. Humans across Asia, Africa, and North America have traditionally chewed some species of sticks as a form of oral hygiene, which are often called tools with no hesitations (e.g., Wu et al., 2001) despite the similarity to behaviors observed in other species. Although such papers are not oriented toward cognitive psychology, but toward the dental hygiene itself, and as such do not define what is a tool from their perspective, this shows that the behavior meets commonplace understanding of tool use and is taken for granted. We know of no papers directly comparing stick chewing behavior in humans with other animals, but if human cases are considered tool use, dogs chewing sticks should be given the same status. The behavior is most familiar in dogs, but stick chewing, especially in the teething stage of development, may be widespread and warrants direct comparative research. In all cases, the same overt behavior should be given the same status as tool use or not. Figure 2 depicts side-by-side images of stick chewing behavior in four species. Despite the similarity of the overt behavior, only the human case is typically identified as tool use.

## Discussion

Some readers might want tool use to require an intentional component (though as noted for hardwired, inflexible tool use, this remains difficult to interpret) and some might also argue dogs using sticks for these functions do not have such an intention. Classifying stick chewing in dogs as tool use might, to those readers, seem to be an overly generous interpretation. We instead emphasize the opposite implication, not that dogs engaged in stick chewing are displaying higher-order cognitive processing, but that tool use as such can occur without positing complex reasoning abilities and may be more common than is often assumed. Recent reviews have questioned the intelligence long assumed to be required for tool use in its simplest form, proposed that it may be more widespread than typically believed, or argued that tool use should be understood in a broader context of construction behavior (e.g., Hansell and Ruxton, 2008; Guillette and Healy, 2015; von Bayern et al., 2020). Further, technology such as camera traps enable large data sets of high-quality observation of species that would be painstaking by standard field observation. Such increased monitoring may allow observation of relatively infrequent tool use in species not typically considered in discussions of tool use, as was the case with puffins (Fayet et al., 2020). This case may not stand alone, and given significantly more monitoring and opportunities to observe naturalistic behavior among species less often targeted for cognitive research, we may see more abilities and capacities previously unrecognized, most particularly in remote, small, and understudied species. If dogs chewing sticks during teething is included in the narrow definition of tool use, it may likewise open the door for numerous other overlooked cases of tool use throughout the animal kingdom, most obviously stick chewing broader species, and possibly other self-maintenance behaviors such as self-scratching on detached materials on the ground (and thus detached from their environment). We encourage readers to think critically about object-oriented behaviors they have seen and revisit whether even commonplace behaviors may be overlooked instances of tool use, especially to consider possible tool use even in contexts without clear analogies to the researchers themselves. We suggest that such greater scrutiny alongside increased monitoring may reveal tool use to be significantly more common than often assumed.

That said, variation in the frequency and flexibility of tool use observed in the wild and in experimental studies can still provide some of the most direct and powerful tests into the evolution of complex physical reasoning abilities. If indeed tool use is more ubiquitous than has often been assumed in previous literature, it may open the door to promising new avenues of study in tool use behavior. More specifically, studies on the factors that contribute to flexible and variable modes of tool use compared to similar but rigid and structurally fixed tool use may shed light on which contexts favor the evolution of higher-order physical reasoning. Tool use in itself may be more common than previously expected, but this suggests that more factors are at play driving flexible expression of tool use behavior and physical reasoning abilities, which themselves warrant detailed further study. Tool use may not be best characterized through simple presence/absence, but along a spectrum of complexity. This spectrum of complexity can be studied in each case of tool use to better understand the relevant evolutionary forces that select for higher-order physical reasoning as opposed to tool use propensity in general. This spectrum can range from hardwired, inflexible tool use, through to conditioned responses without an underlying causal understanding, to trial-and-error learning and innate propensities including refinement of tool choice and manipulation technique, and finally to flexible tool use with a deeper understanding of the direct physical potentialities of tools. We tentatively suggest that the case of dogs chewing sticks may fall into the third category, if it involves an innate propensity to chew sticks as a response to teething pain (or in general as a tooth hygiene mechanism) and involves refinement of stick choice and object manipulation technique, which should be directly investigated. We emphasize these categories are not rigid divisions, but instead may represent a continuous spectrum of cognitive complexity required for kinds of tool use, which can help suggest a research program aimed at testing where tool use in other animals fall in this spectrum as a way to study the evolution of the underlying complex cognition itself.

## Future Directions

Although relief of tooth pain during teething and oral hygiene benefits are reasonable and consistent hypotheses with direct human analogies, evidence for these potential benefits of dog stick chewing are necessary. Most importantly, a direct benefit to the animal must be empirically established through concrete data. This can be done by measuring the impact of chewing hard objects on pain relief during teething, in dogs as well as other species (including humans). More specifically, behavioral indicators of pain as well as close measurement of teething stages (teeth rupture, blood, and gum swelling) can be correlated with frequency of stick chewing. It should be noted that chewing due to teething pain is a standard explanation for chewing by puppies, and similarly hard teething rings and other objects given to chew are among the most common treatments for human teething pain thought to be effective by overwhelming sensory receptors (Ashley, 2001; McIntyre and McIntyre, 2002; Tsang, 2010); however, direct empirical data are needed. In suggesting the kinds of objects to treat infant teething pain, Rousseau (1762) interestingly claimed that dog and wolf puppies make use of softer objects such as sticks and rags rather than iron and bones during teething, and even suggested likewise to provide infants with tree branches to chew. However, we know of no studies empirically testing Rousseau’s assertions. In this vein, possible tool selection seems to be a promising and important direction of study.

Regarding tooth and gum health, comparisons should be conducted between frequent stick chewers and infrequent stick chewers on overall gum health late in life, as well as within-subject comparisons on frequency of stick chewing following consumption of different foods. Function–structure interface analysis should also be conducted (as mentioned earlier) to tease apart alternate motivations for stick chewing depending on the context and form of the behavior (e.g., Pelletier et al., 2017; Cenni et al., 2020), where stick chewing may not be functional in all cases and fine-grained analysis of behavioral patterns can therefore help distinguish those that are true tool use from those that on the surface may appear similar.

Future research should additionally study the amount of planning (e.g., searching for tools or acquiring a tool for later use) and the degree of tool selection involved in the specific case of dog stick chewing as well as the frequency of stick chewing during teething in other species. It will be interesting to see whether robust stick preferences, tool manufacture (for example, breaking sticks off larger branches, or peeling bark before chewing), and tool retention through time are frequently observed or vary by context. However, caution should be taken before assuming that these behaviors represent higher-order thinking and planning. Experiments aimed at testing consistency both within and across individuals about preferences across contexts and time, as well as experimentally altering the sensory properties of potential tools (smell, texture, and appearance), can provide a way to test the cognitive mechanisms involved in dogs chewing sticks.

Comparisons with other canids and broader families will be one of the most powerful and important areas of investigation on the evolution of this form of tool use if it is confirmed in dogs. As in Figure 2, a brief internet search on many species yields photos of adolescents chewing on sticks, suggesting the possibility of more widespread occurrence, but these are anecdotal and we strongly encourage wide surveys of stick chewing in other animals. Such phylogenetic research, both observational and experimental, will be essential in investigating more precisely the form, function, and evolution of stick chewing across species. It will be fascinating to conduct comparative tests on the level of selectivity and degree of planning if indeed stick chewing is widespread and can involve such cognitive capacities, providing a straightforward way to test hypotheses about cognitive development and the evolution of tool use propensity across phylogenies.

Finally, we reiterate the importance of researchers paying closer attention to seemingly unremarkable object manipulation in other species that also may be overlooked instances of animal tool use. Dog (and other animals) stick chewing being an overlooked case of tool use does not necessarily indicate that there are other similarly overlooked cases, but the fact that a common behavior in nearly all adolescents (and many adults) in the species living closest to humans has thus far been given almost no attention is strong reason to think more critically about other cases that may exist. Greater attention to self-scratching with detached objects in species which are known to rub against fixed objects for bodily maintenance, attention to object manipulation in forms and contexts not as easily analogized to humans, as well as wider surveys of species that are less frequently observed by cognitive psychologists may similarly reveal tool use that has gone unreported due largely to a lack of attention.

## Conclusion

In sum, tool use may be significantly more common in non-human animals than is typically assumed, as exemplified by the ubiquitous but possibly underappreciated case of dogs chewing sticks during teething. Dogs chewing sticks fits major definitions of tool use, despite the relative lack of research attention and interest in the behavior. Further work is needed to understand the exact cognitive factors and functions involved in dog stick chewing behavior, but the behavior may suggest that the sharp divide seen in many studies between tool use and interaction with the environment may be blurrier than most papers would suggest and that tool use should be instead conceptualized as a spectrum of physical reasoning skills. A common behavior familiar to almost all dog owners may in fact be an example of one of the most indicative and compelling behaviors in the field of animal cognition.

## Data Availability Statement

The original contributions presented in the study are included in the article/Supplementary Material, further inquiries can be directed to the corresponding author.

## Author Contributions

JB and SY conceptualized, drafted, and revised the manuscript. Both authors contributed to the article and approved the submitted version.

## Conflict of Interest

The authors declare that the research was conducted in the absence of any commercial or financial relationships that could be construed as a potential conflict of interest.

## Footnotes

1 This also invites the possibility of dogs bringing sticks to their owners as social tool use used to trigger play, a possibility we do not explore here but invite for future research.
