# Supplementary material for: Dog Stick Chewing: An Overlooked Instance of Tool Use?
Source: Front Psychol. 2021 Jan 13;11:577100. doi: 10.3389/fpsyg.2020.577100 (PMC7838539; doi:10.3389/fpsyg.2020.577100)
Supplement: Supplementary file 1 [file Data_Sheet_1.docx]

Supplementary Material:

S1: Videos of dogs chewing, but not eating, sticks

<https://www.youtube.com/watch?v=hYciOhZG34s>

<https://www.youtube.com/watch?v=0NH0_-TNyfA>
